# Exploration of morbidity, suicide and all-cause mortality in a Scottish forensic cohort over 20 years

**DOI:** 10.1192/bjo.2020.40

**Published:** 2020-06-18

**Authors:** Cheryl Rees, Lindsay Thomson

**Affiliations:** Division of Psychiatry, University of Edinburgh, UK; Forensic Psychiatry, University of Edinburgh; The State Hospital, Scotland; and The Forensic Mental Health Managed Care Network, Scotland, UK

**Keywords:** Forensic mental health, premature mortality, unnatural death, morbidity

## Abstract

**Background:**

Premature mortality among patients experiencing forensic care is high. This paper examines the morbidity and mortality of all Scottish high secure patients in 1992/1993 and followed up 20 years later through the context of recovery.

**Aims:**

To explore morbidity and delineate which patients are at greatest risk of premature mortality. To assess the extent of suicide and unnatural deaths. To establish which factors, if any, appear protective.

**Method:**

Health and mortality data were extracted from national data-sets and death categorised as premature or post-expected age. Standardised mortality ratios were calculated to explore natural, unnatural and suicide deaths with Cox regression conducted to explore baseline demographics and premature death.

**Results:**

During a mean follow-up of 21.1 years, 36.9% (*n* = 89) died, at an average age of 55.6 years. Of these, 70.8% (*n* = 63) died prematurely. Men lost on average 14.9 years and women 24.1 years of potential life. Five lives (5.6%) were lost by suicide and three (3.4%) by unnatural means.

**Conclusions:**

In contrast to other mainstream and forensic cohorts, high rates of suicide and accidental deaths were not apparent. Risk of premature mortality is high. A greater focus upon physical health by community and in-patient services is essential.

## Background

The elevated mortality risk associated with a diversity of mental disorders when contrasted with the general population is an accepted finding.^1–3^ The number of years of life lost in relation to all-cause mortality varies from 7 to 24 depending on the nature of the condition.^2^ Substance use disorder (SUD) conveys the highest potential number of years lost (9–24); however, this is closely followed by the ranges for personality disorders (13–22 years), schizophrenia (10–20 years) and bipolar disorder (9–20 years) demonstrating a psychophysiological influence (which we define as a physiological response mediated by biochemical pathways to psychological distress) upon morbidity/mortality beyond the physical impact of illicit substances.

In relation to dual diagnosis the risk of death among individuals with severe mental illness (SMI) and concurrent SUD is in excess of those with SMI alone.^4^ Evidence suggests all–cause mortality rates for individuals with schizophrenia may also be increasing,^1,5,6^ but high rates of suicide, particularly after discharge from psychiatric hospital admissions^7^ and unnatural deaths^8^ do not entirely account for the observed disparity in mortality compared with the general population.^1^

## All-cause mortality in forensic settings

Although research primarily focuses on mainstream services, higher rates of all-cause mortality have also been reported within prison settings where the burden of mental health is also in excess of general population comparisons,^9^ with mortality risk further compounded by the high prevalence of SUD reported among prisoners.^10^ Similar high rates of all-cause mortality are apparent among the forensic psychiatry literature.^11–13^ In 2011, Clarke *et al*^12^ noted the deaths of 9.6% of their cohort (*n* = 595) over a maximum 20-year follow-up, Fazel *et al*^11^ reported 29.9% of their population (*n* = 6520) dying over a mean of 15.6 years and Coid *et al*^13^ found 4.9% of their cohort (*n* = 409) died over a mean of 6.2 years. Although they report differing mortality rates they all exhibit high rates of suicide, 32%, 22.7% and 50% of the reported deaths, respectively. In addition, Fazel *et al*^11^ noted the death of 14.2% of their cohort and Clarke *et al*^12^ 22.8% of deaths from accidental/unnatural causes.

There is limited literature regarding morbidity/mortality among forensic patients who represent a specific subset of individuals experiencing SMI.^14^ Findings from mainstream populations cannot be safely generalised because of differing recovery and treatment pathways which, in the case of forensic patients, require to be balanced against the risk to the public.^15^ To address this and from within the context of recovery, a cohort from The State Hospital, Carstairs, the high secure hospital for Scotland and Northern Ireland, has been explored 20 years from their involvement in The State Hospital Survey.^16^ As part of this follow-up morbidity and mortality has been examined to explore influencing factors at a local level.

## Aims

The aims of this paper are to:

- explore morbidity among this group;
- delineate which patients are at greatest risk of premature mortality;
- assess the extent of suicide and unnatural deaths;
- establish which factors, if any, appear protective to cohort members.

## Method

### Cohort

The State Hospital Survey^16^ identified a whole population cohort of 241 patients (male *n* = 213, mean age 36 years, female *n* = 28, mean age 32 years) resident in the high secure State Hospital, Carstairs, at some point between 25 August 1992 and 13 August 1993. Detention was under civil (*n* = 92, 38.2%) and criminal procedures (*n* = 149, 61.8%). Baseline data were collected from case notes and clinical interview. At baseline, the mean lifetime psychiatric stay was 9.3 years (range: 0.08–45). Following the baseline study the mean high security admission was 6.8 years (median 4.3, range: 0.09–22.4). Individuals who were subject to restrictions on discharge, excluding prison transfers (*n* = 81), experienced a mean 9.4 years (median 6.1, range: 0.15–22.4) in high security. Non-restricted individuals and individuals who were prison transfers (*n* = 160) spent a mean of 5.4 years (median 3.5, range: 0.09–22.4) in high security. Additional detailed cohort characteristics have been reported elsewhere.^16^

In 2014 follow-up was initiated with clinical and morbidity data collected for a mean of 19.2 years and mortality data only, for a mean of 21.1 years (median 25.1, range: 0.61–25.4) with 5093.61 person-years at risk (PYAR).

The authors assert that all procedures contributing to this work comply with the ethical standards of the relevant national and institutional committees on human experimentation and with the Helsinki Declaration of 1975, as revised in 2008. The study was approved by South East Scotland Regional Ethical Committee 01, reference 15/SS/0015. Supplemental approvals and the wider study protocol are described elsewhere.^17^ Written informed consent was obtained from all living participants.

### Data sources

The Electronic Data Research and Innovation Service (eDRIS) utilised the cohort's unique Scottish Community Health Index (CHI) numbers (CHI is a population register used for healthcare purposes) to search the data-sets of National Services Scotland (NSS), the Scottish Health data controller. NSS provided Scottish morbidity/mortality information and emigration data. The National Health Service Central Register (NHSCR), using full name and date of birth, supplied UK mortality information and indicated general practitioner registration out with Scotland. Robust mortality status information was unavailable for seven individuals traced to Northern Ireland and one individual who was overseas therefore these eight male participants are classed as mortality status unknown.

NSS provided date of death and ICD-9/10 codes for causes of death. NHSCR corroborated that data and supplied all death certificate information, identified deaths undetected by NSS and deaths within the rest of the UK. NSS provided ICD-9/10 codes recorded during general hospital in-patient and day-case admissions (Scottish Morbidity Record 01), event duration, partial date (month/year) and admission type with data requested for all deceased and consented individuals.

Years of birth for the deceased cohort were applied to the expectation of life, by gender and selected age, Scotland, 1861 to 2011 table.^18^ Deaths were categorised as premature; defined as dying before the age listed based on year of birth/closest period or as post-expected, where death occurred at any point beyond the specified age. Using indirect standardisation^19^ we calculated standardised mortality ratios (SMR) and 95% CI comparing the risk of death among the cohort with the risk of death within the Scottish population.

### Data analysis

We used Cox regression analysis to examine variation in risk of premature death over time according to baseline characteristics. The time in the study ran from 1 September 1992 (or recruitment to baseline study) until 31 December 2017 or date of death. Cases of individuals experiencing death post-expected age were filtered from analysis. The eight individuals with mortality status unknown were right censored^20^ with time in study as recruitment until discharge from The State Hospital when they were lost to follow-up. Independent variables were based upon previous literature and where preliminary analysis yielded statistically significant results.

Kaplan–Meier plots were constructed for each covariate to assess the assumption of proportional hazard. Where the assumption was violated a time-dependent covariate was included. Cox regression models were constructed for each covariate while controlling for age at baseline. All analysis were conducted using SPSS version 22.^21^

## Results

### Mortality

Eighty-nine individuals (36.9%) were deceased as of 31 December 2017 providing an all-cause crude death rate (CDR) of 1747/100 000 PYAR (95% CI 1403–2150). At point of death: 51.7% (*n* = 46) were resident in the community, 30.3% (*n* = 27) within low secure/open wards, 15.7% (*n* = 14) in high security and 2.2% (*n* = 2) were detained in prison.

Mean age of death was 55.6 years (range: 30.5–84.7). Seventy-seven (36.2%) men died and 12 (42.9%) women at average ages of 56.6 years (range: 30.5–84.7) and 48.9 years (range: 36.2–66.1) respectively.

Table 1 outlines mortality status by primary diagnosis. As detailed in Table 2, 52 (67.5%) men died prematurely, and 11 (91.7%) women died prematurely. The mean years of potential life lost were 14.9 years (range: 0.09–35.7) for the men and 24.1 years (range: 5.2–35.8) for the women (10 women; one woman was born, lived primarily and died overseas). Excluding prison transfers, 22.2% (*n* = 18/81) prematurely deceased individuals were subject to restrictions at baseline (specific mortality rate 1052/100 000 PYAR, 95% CI 623–1663) with 28.1% (*n* = 45/160) non-restricted individuals having prematurely died (specific mortality rate 1330/100 000 PYAR, 95% CI 970–1780).

**Table 1:** Mortality status by primary diagnosis at baseline

	Status at follow-up *n* (%)			Total *n* (%)	Specific mortality rate^a^ (95% CI)
	Alive	Dead	Unknown		
Primary diagnosis (baseline)					
Schizophrenia	94 (55.6)	71 (42.0)	4 (2.4)	169 (70.1)	2007 (1567–2532)
Depression	4 (66.7)	1 (16.7)	1 (16.7)	6 (2.5)	847 (21–4718)
Mania	1 (50.0)	1 (50.0)	0 (0)	2 (0.8)	2773 (70–15 451)
Antisocial personality disorder	6 (46.1)	6 (46.1)	1 (7.7)	13 (5.4)	2367 (869–5152)
Alcoholism/drug misuse	3 (60.0)	2 (40.0)	0 (0)	5 (2.1)	1817 (220–6566)
Intellectual disability	27 (84.4)	5 (15.6)	0 (0)	32 (13.3)	657 (213–1532)
Organic brain syndrome	3 (75.0)	1 (25.0)	0 (0)	4 (1.7)	1109 (28–6181)
Undiagnosed psychiatric illness	6 (66.7)	1 (11.1)	2 (22.2)	9 (3.7)	544 (14–3029)
None	0 (0)	1 (100)	0 (0)	1 (0.4)	–^b^
Comorbid diagnosis (baseline)					
Antisocial personality disorder^c^	39 (59.1)	26 (39.4)	1 (1.5)	66 (28.9)	–
Heavy alcohol use/misuse	72 (61.5)	40 (34.2)	5 (4.3)	117 (48.5)	–
Substance use	73 (64.6)	37 (32.7)	3 (2.7)	113 (46.9)	–
Total *n* (%)	144 (59.8)	89 (36.9)	8 (3.3)	241 (100)	–

a. Specific mortality rate per 100 000 person-years at risk.

b. Not calculated due to small group size/person-years at risk.

c. Antisocial personality disorder was the only comorbid personality disorder noted at baseline.

**Table 2:** Death categories by gender

Death Category	Men, deceased (years)		Women, deceased (years)	
	*n* (%)	Mean age (range)	*n* (%)	Mean age (range)
Post-expected age	25 (32.5)	69.1 (61.3–84.7)	1 (8.3)	66.1 (66.1)
Premature death	52 (67.5)	50.6 (30.5–64.3)	11 (91.7)	47.4 (36.2–63.5)
Total	77 (100)	56.6 (30.5–84.7)	12 (100)	48.9 (36.2–66.1)

The SMRs by gender are detailed in Table 3. The SMR for all-cause deaths (SMR = 397, 95% CI 321–487) indicates a mortality rate almost four times that observed within the Scottish population. The ratio for the males (SMR = 297, 95% CI 236–370) returns a threefold increase in mortality whereas the females exhibit an SMR (SMR = 1000, 95% CI 542–1700) ten times the population rate. A high proportion of deaths (91.0%) occurred as a result of natural causes (SMR = 401, 95% CI 321–496).

**Table 3:** Standardised mortality ratios (SMR) by nature of death and baseline demographics

	Observed deaths	Expected	SMR	Lower CI 95%	Upper CI 95%	*P*
All cause						
Overall	89	22.4	397	321	487	<0.001
Male	77	25.9	297	236	370	<0.001
Female	12	1.2	1000	542	1700	<0.001
Natural						
Overall	81	20.2	401	321	496	<0.001
Male	71	22.9	310	244	389	<0.001
Female	10	1.1	909	462	1620	<0.001
Unnatural						
Overall	3	0.8	375	95	1021	n.s.
Male	2	1.1	180	30	600	n.s.
Female	1	0.04	2500	130	12330	<0.05
Suicide						
Overall	5	0.8	625	229	1385	0.001
Male	4	1.5	267	85	643	n.s.
Female	1	0.05	2000	100	9864	<0.05
Age bands, years						
15–44	18	3.7	487	297	754	<0.001
45–64	52	12.8	406	307	529	<0.001
≥65	19	5.9	322	200	494	<0.001
*Diagnosis*						
Schizophrenia						
All	71	15.8	449	354	563	<0.001
Male	62	18.2	341	263	434	<0.001
Female	9	0.9	1000	488	1835	<0.001
Intellectual disability						
All	5	2.7	182	67.9	411	n.s.
Male	5	3.4	147	53.9	326	n.s.
Female	0	0.05		.	.	.
Dissocial personality disorder						
All	6	1.7	361	147	752	<0.05
Male	4	1.8	224	71	539	n.s.
Female	2	0.18	1111	186	3671	<0.01

n.s., not significant.

Suicide was defined by explicit codes; E950–959 (ICD-9) and X60–X84 (ICD–10). In addition, National Records Scotland include ‘event of undetermined intent’ among probable suicides. There were five suicides representing 5.6% of the 89 deaths (CDR 98/100 000 PYAR, 95% CI 32–229). Suicide mortality (SMR 625, 95% CI 229–1385) was six times the population rate. Accidental deaths occurred in three individuals (3.4%) (SMR = 375, 95% CI 95–1021) at almost four times the population rate. Re-categorising substance misuse deaths (*n* = 4, 4.5%) as accidental raises unnatural mortality to almost nine times (SMR = 875, 95% CI 382–1731) the population rate.

### Cause of death

Underlying cause of death was assessed using ICD-9/10 classifications and categorised as premature/post-expected age as detailed in Table 4. Premature death occurred in 63 (70.8%) people. The primary cause of premature death (*n* = 20, 31.7%) was respiratory disease/cancer, with circulatory disease/events (*n* = 12, 19.0%) forming the next largest group with other cancers responsible for 11.1% (*n* = 7). There were no significant differences between premature and post-expected age deaths by cause.

**Table 4:** Underlying cause, gender and death categorisation of deceased group

Cause of death (underlying)	Death category			
	Premature, *n* (%)	Post-expected age, *n* (%)	All deaths, *n* (%)	*P*^a^
Respiratory disease (including bronchopneumonia)	11 (17.5)	6 (23.1)	17 (19.1)	n.s.
Respiratory cancer	9 (14.3)	6 (23.1)	15 (16.9)	n.s.
Circulatory disease/event	12 (19.0)	7 (26.9)	19 (21.3)	n.s.
Cancer (other)	7 (11.1)	4 (15.4)	11(12.4)	n.s.
Infection (including hepatitis, HIV and sepsis)	5 (7.9)	1 (3.8)	6 (6.7)	n.s.
Other natural causes, for example renal failure, peritonitis	4 (6.3)	1 (3.8)	5 (5.6)	n.s.
Drug/alcohol dependence	4 (6.3)	0 (0)	4 (4.5)	n.s.
Suicide/undetermined intent	5 (7.9)	0 (0)	5 (5.6)	n.s.
Traumatic death (unnatural)	3 (4.8)	0 (0)	3 (3.4)	n.s.
Ill-defined or undetermined cause (natural)	3 (4.8)	1 (3.8)	4 (4.5)	n.s.
Total	63 (100)	26 (100)	89 (100)	n.s.

n.s., not significant.

a. Pearson's χ^2^-test (Fisher's exact test): premature/post-expected age.

As noted there were *n* = 5 suicides. For these people baseline primary diagnoses were mixed: three had schizophrenia, one had antisocial personality disorder (ASPD) and one alcoholism. One had comorbid ASPD. At baseline four reported a history of heavy/abusive alcohol misuse and all confirmed polysubstance drug use. At death, two were under mental health legislation, four lived in the community and one in a low secure ward. All accidental deaths occurred in the community, two because of assault and one asphyxiation with food.

### Statistical analysis

Cox regression analysis (Table 5) suggests that men detained under civil provisions at baseline are more likely to die prematurely than those detained under criminal provisions. Males experience a lower risk of premature death and higher survival than females. Diagnosis of intellectual disability at baseline is associated with lower hazard and increased survival. Being female and having a comorbid diagnosis of ASPD significantly increased the likelihood of premature death, a finding not replicated in the males. In general, and for the males, receiving antipsychotic medication by depot injection was associated with higher hazard and shorter survival. Substance misuse at baseline (alcohol misuse or illicit drug use) was significantly associated with an increased hazard of premature death both overall and among males.

**Table 5:** Hazard ratios (95% CI) for premature death by baseline demographics, adjusted for age at start of study

	Adjusted hazard ratio (95% CI)^a^	*P*	Unadjusted absolute risk reduction (95% CI)^b^
Mental Health (Scotland) Act 1984			
Civil detention (all)	1.80 (1.00–3.23)	0.05	0.12 (–0.24 to 0.01)
Civil detention (female)	0.57 (0.17–1.96)	n.s.	0.13 (–0.23 to 0.50)
Civil detention (male)	2.13 (1.17–3.88)	0.01	−0.15 (–1.67 to –0.07)
Criminal detention	1	–	Reference
Sex			
Female	0.41 (0.18–0.96)	0.04	−0.13 (–0.33 to 0.07)
Male	1	–	Reference
Any intellectual disability diagnosis			
Intellectual disability diagnosis (all)	0.27 (0.08–0.87)	0.03	0.20 (0.07 to 0.33)
Intellectual disability diagnosis (female)	0.04 (0–333.2)	n.s.	0.44 (0.25 to 0.63)
Intellectual disability diagnosis (male)	0.31 (0.10–1.01)	0.05	0.17 (0.04 to 0.31)
No Intellectual disability diagnosis	1	–	Reference
Schizophrenia diagnosis			
Schizophrenia diagnosis (all)	1.98 (0.92–4.28)	n.s.	–0.14 (–0.26 to –0.02)
Schizophrenia diagnosis (female)	1.34 (0.36–5.06)	n.s.	–0.11 (–0.50 to 0.27)
Schizophrenia diagnosis (male)	2.27 (0.95–5.45)	n.s.	–0.15 (–0.27 to –0.02)
No schizophrenia diagnosis	1	–	Reference
Comorbid diagnosis dissocial personality disorder (DPD)			
Comorbid DPD (all)	1.07 (0.49–2.35)	n.s.	–0.05 (–0.19 to 0.09)
Comorbid DPD (female)	8.36 (1.38–50.73)	0.02	–0.37 (–0.81 to 0.06)
Comorbid DPD (male)	0.97 (0.53–1.78)	n.s.	0.01 (–0.15 to 0.13)
No comorbid DPD diagnosis	1	–	Reference
Medication			
Depot neuroleptics at baseline (all)	1.98 (0.99–3.93)	0.05	–0.17 (–0.29 to –0.05)
Depot neuroleptics at baseline (female)	2.34 (0.51–10.87)	n.s.	–0.22 (–0.60 to 0.15)
Depot neuroleptics at baseline (male)	3.69 (1.11–12.22)	0.03	–0.16 (–0.29 to –0.04)
No depot neuroleptics	1	–	Reference
Substance misuse^c^			
Drug misuse recorded (all)	3.71 (1.30–10.58)	0.01	–0.14 (–0.27 to –0.02)
Drug misuse recorded (female)	3.5 (0.41–29.54)	n.s.	–0.25 (–0.62 to 0.13)
Drug misuse recorded (male)	3.44 (1.02–11.61)	0.05	–0.13 (–0.26 to 0.01)
No recorded drug misuse	1	–	Reference

a. Adjusted for age at baseline.

b. Compared with appropriate ‘control’ group. Negative value, absolute risk increase.

c. Any drug misuse or heavy alcohol/misuse reported at baseline.

### Morbidity

Data relating to Scottish general hospital in-patient admissions for the 89 deceased and 66 consented participants were obtained from NSS (*n* = 154 as one participant died following consent). A nil return was obtained if the individual had never had a general hospital in-patient admission in Scotland between baseline and 31 December 2014/date of death. Data were acquired for 115, with a further 39 individuals receiving a nil return. Table 6 reports the mean number of unique ICD-10 codes allocated for each ICD-10 block. Table 7 outlines the number of individuals in receipt of an ICD-9/10 classification by ICD-10 block description and Table 8 the number of days spent as a general hospital in-patient.

**Table 6:** Mean number of ICD-10 codes allocated for each ICD-10 block

ICD-10 Blocks	Alive (*n* = 65)		Premature death (*n* = 63)		Post-expected age (*n* = 26)		Total (*n* = 154)		*P*^a^
	Mean	s.d. (min–max)	Mean	s.d. (min–max)	Mean	s.d. (min–max)	Mean	s.d. (min–max)	
A/B –Certain infectious and parasitic diseases	0.12	0.4 (0–2)	0.44	1.0 (0–6)	0.23	0.6 (0–2)	0.27	0.8 (0–6)	0.027
C/D – Neoplasms	0.14	0.6 (0–3)	0.33	0.7 (0–3)	0.54	0.8 (0–2)	0.29	0.7 (0–3)	0.009
D – Diseases of blood and blood forming organs/certain disorders of immune mechanism	0.12	0.4 (0–2)	0.16	0.4 (0–1)	0.19	0.5 (0–2)	0.15	0.4 (0–2)	n.s.
E – Endocrine, nutritional and metabolic diseases	0.18	0.5 (0–2)	0.27	0.6 (0–3)	0.46	0.7 (0–2)	0.27	0.6 (0–3)	n.s.
G – Diseases of the nervous system	0.14	0.5 (0–3)	0.43	0.8 (0–3)	0.54	0.9 (0–4)	0.32	0.7 (0–4)	0.005
H – Diseases of the eye and adnexa/ear and mastoid process	0.14	0.5 (0–2)	0.1	0.4 (0–2)	0.19	0.5 (0–2)	0.13	0.4 (0–2)	n.s.
I – Diseases of the circulatory system	0.26	0.6 (0–2)	0.59	1.6 (0–11)	1.23	1.3 (0–4)	0.56	1.2 (0–11)	n.s.
J – Diseases of the respiratory system	0.48	1.0 (0–4)	1.44	2.1 (0–9)	1.42	2.0 (0–8)	1.03	1.8 (0–9)	0.002
K – Diseases of the digestive system	1.25	2.2 (0–9)	1.32	1.9 (0–10)	0.65	1.2 (0–5)	1.18	1.9 (0–10)	n.s.
L – Diseases of the skin and subcutaneous tissue	0.25	0.6 (0–3)	0.37	0.7 (0–3)	0.19	0.5 (0–2)	0.29	0.7 (0–3)	n.s.
M – Diseases of the musculoskeletal system and connective tissue	0.09	0.3 (0–2)	0.32	0.9 (0–5)	0.31	0.8 (0–3)	0.22	0.7 (0–5)	n.s.
N – Diseases of the genitourinary system	0.38	0.8 (0–4)	0.41	1.3 (0–8)	0.85	1.9 (0–8)	0.47	1.3 (0–8)	n.s.
O/P – Pregnancy childbirth and the puerperium/Perinatal period)	0	0 (0)	0.02	0.1 (0–1)	0	0 (0)	0.01	0.1 (0–1)	n.s.
Q – Congenital malformations deformations and chromosomal abnormalities	0.03	0.2 (0–2)	0.03	0.3 (0–2)	0	0 (0)	0.03	0.2 (0–2)	n.s.
R – Symptoms, signs and abnormal laboratory findings not elsewhere classified	0.94	1.9 (0–12)	1.32	2.5 (0–14)	1.96	3.0 (0–11)	1.27	2.4 (0–14)	n.s.
S–Y – Injury, poisoning & certain other consequences of external causes (includes V–Y and U codes)	1.31	4.0 (0–22)	4.05	9.0 (0–49)	1.12	1.5 (0–5)	2.4	6.5 (0–49)	0.005
Total ICD–10 blocks	5.8	9.4 (0–54)	11.59	15.6 (0–66)	9.88	9.7 (0–33)	8.86	12.6 (0–66)	0.022

Min–max, minimum–maximum; n.s., not significant.

a. Mann–Whitney *U*-test, alive/premature death.

**Table 7:** Number of individuals in receipt of an ICD-10 classification by block description

	Alive, *n* (%)	Premature death, *n* (%)	Dead (post-expected age), *n* (%)	Total, *n* (%)	*P*^a^^,^^b^ (2 tailed)
ICD-10 blocks/codes (total *n*)	*n* = 65	*n* = 63	*n* = 26	*n* = 154	
Zero in-patient days/day surgery events	19 (29.2)	15 (23.8)	5 (19.2)	39 (25.3)	–
A/B – Infectious diseases	7 (10.8)	16 (25.4)	4 (15.4)	27 (17.5)	0.039
C00 –D48 neoplasms	4 (6.2)	15 (23.8)	10 (38.5)	29 (18.8)	0.006
D – Blood/immune	7 (10.8)	10 (15.9)	4 (15.4)	21 (13.6)	n.s.
E – Endocrine/metabolic/ nutritional disease	9 (13.8)	12 (19.0)	9 (34.6)	30 (19.5)	n.s.
G – Nervous system disease	5 (7.7)	17 (27.0)	10 (38.5)	32 (20.8)	0.005
H – Eye/ear disease	6 (9.2)	4 (6.3)	4 (15.4)	14 (9.1)	n.s.
I – cardiovascular disease	13 (20.0)	18 (28.6)	15 (57.7)	46 (29.9)	n.s.
J – Respiratory disease	17 (26.2)	30 (47.6)	15 (57.7)	62 (40.3)	0.017
K – GI disease	30 (46.2)	32 (50.8)	8 (30.8)	70 (45.5)	n.s.
L – Skin/subcutaneous tissue disease.	10 (15.4)	16 (25.4)	4 (15.4)	30 (19.5)	n.s.
M – Musculoskeletal connective tissue disease	5 (7.7)	10 (15.9)	4 (15.4)	19 (12.3)	n.s.
N – Genitourinary disease	13 (20.0)	12 (19.0)	7 (26.9)	32 (20.8)	n.s.
Any pregnancy-related disorder	0 (0.0)	1 (1.6)	0 (0.0)	1 (0.7)	n.s.
Q – Any congenital/chromosomal etc. disorder	1 (1.5)	1 (1.6)	0 (0.0)	2 (1.3)	n.s.
R – Abnormal clinical findings	28 (43.1)	31 (49.2)	15 (57.7)	74 (48.1)	n.s.
S–Y – Injury, poisoning & certain other consequences of external causes	14 (21.5)	28 (44.4)	12 (46.2)	54 (35.1)	0.008
Obesity	1 (1.5)	2 (3.2)	2 (7.7)	5 (3.2)	n.s.
Type 2 diabetes	5 (7.7)	6 (9.5)	3 (11.5)	14 (9.1)	n.s.
Respiratory cancer	0 (0)	6 (9.5)	6 (23.0)	12 (7.8)	0.013
Non-respiratory cancer	1 (1.5)	4 (6.3)	2 (7.7)	7 (4.5)	n.s.
Alcohol	5 (7.7)	8 (12.7)	2 (7.7)	15 (9.7)	n.s.
Drug	0 (0)	5 (7.9)	0 (0)	5 (3.2)	0.027
Tobacco	2 (3.0)	8 (12.7)	4 (15.4)	14 (9.1)	n.s.

Min–max, minimum–maximum; n.s., not significant.

a. Pearson's χ^2^ test (Fisher's Exact test): Alive/ premature death.

b. ICD-10 blocks/codes.

**Table 8:** Mean days spent within the general hospital in-patient environment

General hospital in-patient days	Alive (*n* = 65)		Premature death (*n* = 63)		Dead (post-expected age) (*n* = 26)		Total (*n* = 154)		
	Mean	s.d. (min–max)	Mean	s.d. (min–max)	Mean	s.d. (min–max)	Mean	s.d. (min–max)	*P*^a^
Total trauma related	0.49	2.1 (0–15)	14.14	71.1 (0–555)	1	2.6 (0–13)	6.16	45.8 (0–555)	0.007
Total urgent days	3.38	8.5 (0–53)	12.4	28.8 (0–197)	8.08	11.7 (0–40)	7.86	20.1 (0–197)	0.011
Total routine days	3.69	11.2 (0–56)	15	39.5 (0–289)	26.27	48.5 (0–164)	12.13	33.7 (0–289)	<0.001
Total in-patient days	7.57	19.5 (0–121)	41.54	133.1 (0–1041)	35.35	51.8 (0–184)	26.16	89.6 (0–1041)	<0.001

Min–max, minimum–maximum.

a. Mann–Whitney *U*-test: alive/premature death.

Examination of the tables presented provides an overview of the physical health of the alive, prematurely deceased and post-expected age deceased groups. No significant differences were observed in terms of mean endocrine, nutritional and metabolic disease diagnoses between the living participants and those who prematurely died. Similarly, no significant differences were observed for diseases of the circulatory system. In relation to respiratory system diseases, a significant difference was observed in mean diagnoses between prematurely deceased (1.44 diagnoses, *P* = 0.002) and living participants (0.48 diagnoses). In terms of ICD-10 blocks S–Y ‘Injury, poisoning & certain other consequences of external causes’, a significant difference was observed between prematurely deceased (4.05 diagnoses, *P* = 0.005) and living participants (1.31 diagnoses). Unfortunately, injuries resulting from self-harm cannot be distinguished from accidental harm. Prematurely deceased individuals received significantly more diagnoses (11.59, *P* = 0.022) compared with consented participants (5.8 diagnoses).

Those who died prematurely spent on average significantly more days as general hospital in-patients in relation to total trauma (14.1 days, *P* = 0.007), urgent (12.4 days, *P* = 0.011) and routine admissions (15, *P* < 0.001) than living participants.

## Discussion

This study investigated morbidity and mortality findings from a 20-year follow-up of a cohort of high secure forensic patients first recruited as a whole population survey and followed through the context of recovery.

### Unnatural and suicide deaths

In stark contrast to previously reported forensic^6,22^ and mainstream psychiatric findings^2,23^ we did not observe exceptional rates of suicide and accidental/unnatural deaths. We described a sixfold (SMR = 625) increase in suicide and an almost fourfold (SMR = 375) increase in unnatural deaths against the general population. In comparison, Clarke *et al*^12^ reported a SMR for suicide of 3231, and for all unnatural deaths of 1898 in relation to a cohort followed for a maximum of 20 years (*n* = 595, 5593 PYAR) after first admission to a medium secure unit. Including the few drug/alcohol deaths within accidental (unnatural) deaths we only returned an almost ninefold increase. No suicide/accidental deaths occurred within high secure care, and only one of our eight suicide/accidental deaths happened within the psychiatric hospital environment. Of the *n* = 31 suicide/open verdicts and accidental deaths reported by Clarke *et al*^12^ almost half (*n* = 15) the preventable deaths occurred within high/medium security and the wider hospital environment.

We considered a number of factors in exploring this finding of a low rate of suicide and accidental deaths within our cohort. First, was the Scottish suicide rate lower than elsewhere in the UK? The 2018 Scottish suicide rate (16.1/100 000 persons) was greater than the comparably reported English rate (10.3/100 000).^24^ Therefore Scotland does not have a naturally low rate of suicide.

Second, was our low rate of suicide reflected in the general adult psychiatry population in Scotland? This was refuted by a historical Scottish population study of discharged, long-term (>1 year) psychiatric in-patients that reported an increased suicide risk 13 times the population rate.^25^

Third, was the diagnostic profile of this Scottish cohort impactful? Unlike other UK regions,^13^ within Scotland offenders with a primary diagnosis of personality disorder generally remain within the criminal justice system. At baseline only 5% received a primary diagnosis of personality disorder whereas 70% attracted a schizophrenia diagnosis. This compares with 25.8% of individuals with personality disorder in a Swedish forensic cohort,^11^ 26.6% in an English medium secure cohort^12^ and 13.5% in an English community cohort.^13^ Only the Clarke et al^12^ study splits suicide between mental illness and psychopathy at 72.2% and 16.6%, respectively, which is reduced in the personality disorder group given that it was 26.6% of their cohort. The different diagnostic profiles may be a factor in explaining our findings but this is not supported by the Clarke et al^12^ study and would not account for the extent of the difference. Suicide rates are generally high among individuals experiencing personality or psychotic disorder^26^ with risk of accidental death higher for personality disorder compared with schizophrenia.^8^ Contextualising our suicide deaths within our diagnostic profile we observe a similar pattern; 60% of people who died by suicide had schizophrenia at baseline, one person had ASPD and another alcoholism. A total of 80% were male and the same proportion had a history of heavy/abusive alcohol use, all displayed polysubstance drug misuse, known risk factors for non-natural death.^26^ That description differs from the overall cohort, 47% of whom reported substance misuse at baseline and 29% had comorbid ASPD.

Fourth, does the year the cohort was established or the length of follow-up influence findings? Compared with other studies^11–13^ our cohort was followed for the longest time therefore increasing rather than decreasing suicide likelihood. Alternatively, have more recently established cohorts observed behavioural change, making suicide/accidental in-patient death more likely? Only one suicide has been noted in our cohort source hospital (The State Hospital) since 1996 (email from Health Records, The State Hospital, tsh.Health_Records@nhs.net, November 2019).

Finally, is engagement with the Scottish forensic mental health system protective against suicide or accidental death? An examination of suicides (*n* = 14) occurring within high secure care in Scotland (The State Hospital, 1972–1996)^27^ noted treatment-resistant schizophrenia and having committed violent offences as suicide risk factors. Greater liberalisation of the hospital, increased activities and reintroduction of clozapine were suggested for the suicide reduction observed over time. This continuing developmental process alongside estate renewal may influence our observed low suicide/accidental death rate while patients resided in high secure care. At death two individuals were under mental health legislation/restrictions. Examining CDR figures, restrictions made little difference to the premature death rate. Also considered was if a longer period of contact and mandated relationship with services may protect against suicide; alternatively, lack of autonomy may be a negative influence. Neither was apparent within our findings.

We hypothesise that factors within the Scottish forensic in-patient environment; physical, procedural and/or relational are protective in terms of suicide prevention or in deterring behaviour leading to accidental death and that these may have an ongoing effect on patients’ relationships with services in the community. There may also be organisational factors that reduce avoidable deaths. Scottish forensic services have advantages in terms of size and cohesion, with few independent secure beds, and the strategic lead of the Forensic Network.

### Risk of premature mortality

A large proportion of our cohort died (36.9%) demonstrating an almost fourfold increase (SMR = 397) in all-cause mortality. This represents a slightly higher CDR than reported for forensic services in England/Wales,^22^ mirrors an English general adult community-recruited cohort experiencing psychosis^28^ and is lower than reported internationally.^22^ In contrast, 91% of our cohort died of natural causes four times (SMR = 401) the rate of the general population. Natural deaths within the discharged Scottish general adult psychiatry in-patient population has been reported at SMR = 169^29^ indicating a difference in mortality between Scottish forensic and general adult in-patients.

Overall, 70.8% of deaths were premature and naturally occurring at an average age 55.6 years. Respiratory disease/cancer was the underlying cause of almost a third of premature and 36% of all deaths, with cardiovascular disease related to 19% of premature and 21% of all mortality. Study recruitment occurred during 1992/3 when almost all patients smoked or were passive smokers. Since December 2011, our cohort source hospital, The State Hospital, Carstairs has been entirely smoke free.

Rates of respiratory related deaths, in excess of fourfold the expected level have been noted in an early population study of discharged Scottish general adult psychiatry long-term (>1 year) in-patients^25^ with rates of death related to cardiovascular disease being slightly raised at SMR = 160. A later examination^29^ capturing all discharged general adult psychiatric in-patients reported that cardiovascular disease, despite displaying only a small rise in relative risk (SMR = 170) was responsible for 67% of total mortality and 54% of the total years of potential life lost. A similar English study examining death within a year of discharge of patients with schizophrenia reported SMR of 470, and 250 for respiratory and circulatory disease deaths, respectively, with increased rates from 1999 to 2006.^6^

The cohort of 28 females represents around 50% of Scottish female forensic patients, reported as *n* = 56 in 2004^30^ and *n* = 60 in 2017.^31^ There is an obvious disparity between the male and female groups. Almost all women died prematurely aged under 48 years with mean loss of 24 years of potential life. Their SMR for natural death was three- to fourfold the male rate and that held for females with schizophrenia, rising to fivefold for a primary diagnosis of ASPD. Receiving a comorbid diagnosis of ASPD was also associated with premature death. This suggests that ASPD may have a unique impact on the physical health of women in a manner not reflected in risky behaviour or suicide completion.

There is a lack of published literature regarding gender differences in mortality among patients located within forensic services with papers and reviews generally commenting on the percentage of males within the cohort.^13,22^ Where data does exist,^12^ our results replicate those findings, namely that SMR for death because of natural causes, unnatural causes and suicide are higher for the female than the equivalent male cohort. However, as with those presented here, findings must be interpreted with caution because of the extreme gender imbalance evident within forensic psychiatric services and reflected in small numbers of females in research cohorts.

A similar picture has, however, been observed within mainstream services, specifically in relation to individuals with schizophrenia. A population follow-up of all in-patients admitted with schizophrenia diagnosis from 1980 to 2006 indicated that up to 1992 the SMR for males in relation to all-cause, unnatural and natural deaths exceeded that of females; however, for individuals admitted post 1992 the opposite was observed: females displayed greater all-cause, unnatural and natural SMR.^32^ More recently an American population study reported that female SMR for all-cause mortality exceeded the male equivalent; however, the SMR for unnatural death was higher among males.^5^

Almost 68% of males died prematurely at mean age 50 years. Males detained under civil measures were significantly more likely to die prematurely than those males under criminal procedures. Similarly, males in receipt of depot antipsychotics at baseline were significantly more likely to have died prematurely. It is conceivable that as civil patients transfer to high secure care because of acute morbid positive symptoms and/or locally unmanageable levels of violence or aggression^16^ and by utilising depot medication as a proxy for illness severity, we may be observing those who experienced the severest symptoms and psychological distress, dying prematurely. Specifically, that is, individuals who may be more greatly influenced by psychophysiological factors.

Scottish forensic services acknowledge that female patients represent a heterogeneous group, often more chaotic and challenging with differing needs to male patients,^30^ with higher rates of mortality^33^ and generally poorer outcomes.^34^ Although Scotland lacks female high secure care and exclusively single gender medium secure provision (with some services provided within England), because of the general complexity of female patients, providing appropriate relational security and support can be more important than physical security.^31^ Regardless of shortcomings in the Scottish female forensic estate, the high levels of premature mortality of both genders cannot be overlooked.

Despite spending on average longer as high secure in-patients, with exposure to the health benefits that offers, there was little difference in CDR for those subject to restrictions on discharge and those not. Fazel et al^22^ pointed out that mortality rates among forensic in-patients are high but more closely reflect the general adult psychiatry population than prisoners. They postulate that greater than any factors negatively influencing mortality within forensic environments are the poor lifestyle choices evident among general psychiatric populations that can compound psychotropic medication side-effects. Given our respiratory findings, however, the impact of a heavy smoking environment must be acknowledged. Although poor lifestyle choices have an impact on morbidity/mortality among individuals experiencing SMI again the increased morbidity risks and resultant mortality are not fully explained by behavioural patterns.^35,36^

### Protective factors

Within this cohort some protective factors appeared evident. Although both genders died at early ages the males lived on average almost 8 years longer compared with the females. Males with a primary diagnosis of intellectual disability appeared less likely to die (SMR = 147, 95% CI 53.9–326) and it could be that those patients with intellectual disability received less psychotropic medication and accompanying side-effects. One reason suggested for high rates of morbidity among individuals with mental ill health is the propensity for diagnostic overshadowing to occur. This is when disparities occur in the treatment and diagnosis of physical disorders as a result of misattribution of physical symptoms to mental illness.^37^ Although we interpreted experiencing primary intellectual disability as a protective factor, diagnostic overshadowing has been presented as a particular problem within the intellectual disability population with symptoms related to physical or mental ill health being misattributed to their intellectual disabilitiy.^38^ We suggest that in line with our assertion that engagement with the Scottish forensic mental health system may be protective against unnatural death and death by suicide, for individuals with intellectual disability, location within services almost exclusively under National Health Service operational control may confer advantages to forensic patients with intellectual disability in terms of staffing, their training and support and patient services offered. Higher levels of in-patient and community support to avoid offending and foster appropriate behaviour may also reduce stress and encourage patients with intellectual disability to adopt heathier lifestyles, with a potential reduction in drug and alcohol use. More attention may be paid to their physical health by staff, and general practitioner referrals encouraged and supported, leading to earlier physical health intervention and a reduction in diagnostic overshadowing.

It is also acknowledged that individuals with intellectual disability represent a particularly vulnerable subpopulation within the prison environment, being subject to high rates of mental disorder^39^ and possibly greater risk of suicidal ideation than the general population.^40^ We propose that a contributing factor to this poor outlook is a lack of equivalence, equality and equity for people with intellectual disability within the UK prison system.^41^ Again we propose that our intellectual disability cohort were protected from premature mortality precisely because they were, where appropriate, diverted from the prison environment and supported by specialist forensic psychiatric services within hospital and community settings, designed to promote and provide equality of life experience. Further research is required to address the paucity of literature robustly identifying and exploring the journey of individuals with intellectual disability through services.

### Morbidity

Antipsychotic medications, a reliable mechanism for easing symptoms, reducing distress and therefore enhancing recovery carry with them a diversity of side-effects: activating, sedating and metabolic. Unsurprisingly antipsychotics have been targeted as a possible reason for the premature mortality associated with schizophrenia.^1,42^ Population studies^43^ indicate that individuals with schizophrenia treated with antipsychotics or antidepressants have a lower risk of death compared with individuals not receiving such medications. A primary diagnosis of schizophrenia was applied to 70% of this cohort, and receiving depot neuroleptics was significantly associated with premature death; however, there were no significant differences in mean diagnoses of cardiovascular or endocrine, nutritional and metabolic disease applied to the living participants and the prematurely deceased. What is apparent is the significant difference in terms of respiratory disease, with those dying prematurely receiving more diagnoses. Although undoubtedly the legacy of an era, smoking remains an issue for forensic patients after discharge from controlled in-patient environments.

Those who died prematurely attracted significantly more diagnoses related to injury, poisoning and other external causes; however, these did not translate into high numbers of traumatic deaths or completed suicides. Overall, those dying prematurely received more physical health diagnoses across all listed ICD-10 blocks and spent significantly more days as general hospital in-patients than those who remained alive evidencing the poorer physical state of that group.

Scotland does not have the healthiest national population, indeed the ‘Scottish effect’ is much examined with 17 hypotheses proposed to account for excessive premature mortality.^44^ We suggest that something akin to the ‘Scottish effect’, specifically in terms of biopsychosocial stress and the resultant physiological stress response, is being observed among mainstream and forensic psychiatric populations. High levels of adverse life events observed among Scottish forensic patients^45^ together with the dose–response association observed between adverse life events/psychological distress and a negative impact upon subjective and objective physical health^46^ may be evidenced within our reported cohort. There is increasing physiological evidence of heightened levels of oxidative stress and inflammation within anxiety, depressive, bipolar disorder and schizophrenia.^36,47^ These processes provide mechanistic pathways by which leucocyte telomere length can be shortened. Meta-analysis has evidenced shortened telomere length across a range of psychiatric disorders^36^ with length being an indicator of cell ageing and short length associated with age-related morbidities, for example immune dysregulation, cancer, diabetes and cardiovascular disease.^48^ Interpreting the morbidity and excess mortality observed in cohorts such as this through the lens of the ‘Scottish effect’ may lead to better targeted local interventions.

### Limitations

This study has several limitations. Follow-up physical health information could only be requested for deceased or consented participants. The wider study adopts a gatekeeper approach therefore all cohort members whose gatekeeper denied access because of their inability to provide ‘fair’ consent^49^ or they were not physically/mentally well enough to be approached were excluded. This removed individuals with the greatest physical health needs and/or the severest psychiatric symptomatology. The mortality status of eight individuals remained unknown although of these seven resided in Northern Ireland. We could not locate or engage with appropriate Northern Ireland services to confirm their status or locate their current care team. It proved impossible to access information without consent. Accessing cohort member gatekeepers within England was equally difficult but mortality information moved between NHSCR and their English counterparts. Some individuals with mortality status unknown could have died because of suicide or accidental causes, raising our mortality profile, but it would remain lower than the reported studies. We were also as reliant on the general hospital clinicians accurately recording and/or applying the most appropriate ICD-10 codes as we were of the cohort member and their accompanying staff providing a precise physical health history.

## Data Availability

C.R. had full, ongoing access to data; L.T. had full, ongoing access to data.
